# 4296 Targeting ERG Through Toll-Like Receptor 4 in Prostate Cancer

**DOI:** 10.1017/cts.2020.95

**Published:** 2020-07-29

**Authors:** Ben Greulich, Josh Plotnik, Peter Hollenhorst

**Affiliations:** 1 Indiana University School of Medicine

## Abstract

OBJECTIVES/GOALS: The objective of this research was to learn how the oncogenic transcription factor, ERG, is regulated in prostate cancer. If we could learn how ERG is regulated and which genes are important for its oncogenic phenotype in prostate cells, we could design new therapeutic strategies against ERG, which has proven to be difficult to target. METHODS/STUDY POPULATION: We conducted an shRNA screen in prostate cells to determine candidate genes and pathways that are important for ERG function. To validate the findings of the screen, we performed a variety of cell-based functional assays, including trans-well migration, wound healing, and clonogenic survival assays. To further investigate the mechanism between ERG and the genes revealed by the screen, we performed biochemical and molecular biology experiments such as Western blotting and qRT-PCR for protein and mRNA expression, co-immunoprecipitation assays to determine protein-protein interactions, and chromatin immunoprecipitation (ChIP-qPCR) to determine transcription factor binding to DNA sites. RESULTS/ANTICIPATED RESULTS: The screen revealed that genes involved in the toll-like receptor 4 (TLR4) pathway are important for ERG-mediated migration. We tested the effect of a TLR4 inhibitor on ERG function and observed decreased migration and clonogenic survival exclusively in ERG-positive cells. Expression of pMEK and pERG was reduced when TLR4 was inhibited, which suggests a mechanism in which TLR4 upregulates pMEK, leading to the phosphorylation and activation of ERG. This is supported by functional assays in which cells expressing a phosphomimetic ERG are resistant to the TLR4 inhibitor. We demonstrated that ERG drives the transcription of *TLR4* and its endogenous ligands *HSPA8* and *BGN*. Therefore, ERG can sensitize the cell to TLR4 activation by increasing the number of receptors as well as providing the ligands needed for stimulation. DISCUSSION/SIGNIFICANCE OF IMPACT: This research provides a new therapeutic pathway for treating ERG-positive patients through TLR4 inhibition. This can be beneficial because many patients become resistant to the standard therapy, leaving very few treatment options. TLR4-based therapies could provide an alternative for patients who have developed resistance.

